# Vanishing Tumours

**Published:** 1899-03-11

**Authors:** 


### VANISHING TUMOURS.

At the last meeting of the Harveian Society Mr. D'Arcy Power read a paper on Vanishing Tumours, meaning by the term not ordinary phantom tumours, but true swellings, visible to the eye, apparent to the touch, and capable of exposure by ordinary surgical methods, occurring at any age and in either sex, most often innocent, but sometimes malignant, and yet "vanishing"—that is, passing away under some such simple surgical operation as incision, puncture, or even simple exposure. A considerable number of cases were related in which tumours of very varied character had disappeared either spontaneously or after partial removal or mere exploratory incision. Occasionally such cases are true sarcomata or carcinomata, but much more frequently they are inflammatory swellings of the connective tissue, and they occur, therefore, where connective tissue is abundant in quantity and loose in texture. They are formed slowly and painlessly, and they disappear in like manner; yet being of considerable size, and consisting of cells which are multiplying actively, they may cause pressure symptoms and constitutional disturbances which may make it difficult to distinguish an innocent from a malignant growth. Now, a most interesting point is, What would have become of these tumours if they had been left alone? To this it seems impossible to give any general answer; but what appears clear from the cases related by Mr. Power is that very considerable thickenings and enlargements—tumours, in fact—may become absorbed under the influence of comparatively slight interference with their existing conditions. So far as these tumours are really inflammatory in their origin, which probably is the case in the majority of instances, the explanation of the result may be that tension is relieved and a series of complicated physiological changes are set up which end in resolution; and, although we are ignorant of the nature of these changes, we assume them to be the direct outcome of alterations in the vasomotor and trophic functions. The older surgeons set these changes in action by bleeding, obtaining in this way a local effect by modifying the whole blood pressure, and it is certain that phlebotomy cured many obscure swellings. Now, instead of drawing blood from the arm, we attack the part affected. But, although it is comparatively easy to understand how a simple inflammatory swelling may disappear, it is very difficult to realise, much more to explain, the vanishing of a malignant tumour. Yet there can be but little doubt that sarcomata, and in some very rare cases even carcinomata, do actually disappear; in regard to which point the shrinking of carcinoma of the breast after removal of the ovaries and the contraction of enlarged prostates on removal of the testes must be remembered. Nevertheless, we must not allow the chance of tumours "vanishing" to interfere with the broad surgical principle that they should be removed as early and as completely as possible, and that the more a tumour is considered malignant the earlier and more complete the removal should be. Still, it has happened in certain cases that for various causes such a complete removal has been impossible, and yet by accident or good luck, coupled with some unusual modification of the morbid process, results have been obtained which have been good, though the method adopted has appeared at the time most unsatisfactory. It is well, then, to remember that when from any cause an attempt to remove a tumour has ended in failure, or an exploratory incision has led to an unfavourable verdict, the last word has not always been said. The cases related should not modify our treatment, but they should make us a little careful in our prognosis.

